# Supplementary material for: Understanding and predicting COVID-19 clinical trial completion vs. cessation
Source: PLoS One. 2021 Jul 12;16(7):e0253789. doi: 10.1371/journal.pone.0253789 (PMC8274906; doi:10.1371/journal.pone.0253789)
Supplement: S1 Table — The 40 statistics features, their subcategory and definition. (PDF) [file pone.0253789.s001.pdf]

S1 Table. Summary of all 40 statistics features and their descriptions (definitions)

Feature Subcategory	Feature Name	Description/Definition
Class Label	Status	1 if Terminated, 0 if Completed
Administrative	Industry Collaborator	1 if Main Collaborator Class is industry 0 otherwise
	Number Collaborators	Number of listed collaborators from clinical trial XML
	Number Officials	Number of listed officials from clinical trial XML
	Responsible Party: Investigator	1 if Responsible Party is Investigator or Sponsor-Investigator
	Industry Sponsor	1 if Sponsor class is industry, 0 otherwise
Eligibility	Average Eligibility Words	Average words per eligibility criteria
	Average Exclusion Words	Average words per exclusion criteria
	Average Inclusion Words	Average words per inclusion criteria
	Eligibility Lines	Number of eligibility criteria
	Eligibility Numbers	Number of numbers in eligibility
	Eligibility Words	Number of words in eligibility
	Exclusion Lines	Number of exclusion criteria
	Exclusion Numbers	Number of numbers in exclusion criteria
	Exclusion Words	Number of words in exclusion criteria
	Inclusion Lines	Number of inclusion criteria
	Inclusion Numbers	Number of numbers in inclusion criteria
	Inclusion Words	Number of words in inclusion criteria
	No Eligibility Requirement	1 if trial has no eligibility, 0 otherwise
	Gender Restriction	1 if trial has gender restriction, 0 otherwise
	Age Restriction	1 if trial has age restriction, 0 otherwise
	Healthy Volunteer	1 if trial accepts healthy volunteers, 0 otherwise
Study Design	Random Groups	1 if trial uses random groups, 0 otherwise
	Placebo Group	1 if trial has a placebo group, 0 otherwise
	Uses Blinding	1 if trial uses masking, 0 otherwise
	Number Arms	Number of groups
	Number Sites	Number of sites listed in clinical trial XML
Study Information	Has DMC	1 if trial has DMC, 0 otherwise
	Has Oversight	1 if trial has DMC or FDA regulation, 0 otherwise
	Has Expanded Access	1 if trial has expanded access, 0 otherwise
	FDA Regulation	1 if trial has FDA drug or FDA device regulation, o otherwise
	Main Country: USA	1 if main country is USA, 0 otherwise
	Number Countries	Number of countries listed in clinical trial XML
	No Phase	1 if no phase or phase 0, 0 otherwise
	Phase 1	1 if phase 1 or phase 1/2, 0 otherwise
	Phase 2	1 if phase 2 or phase 1/2 or phase 2/3, 0 otherwise
	Phase 3	1 if phase 3 or phase 2/3, 0 otherwise
	Phase 4	1 if phase 4, 0 otherwise
	Interventional Study	1 if trial is interventional, 0 if observational
