# Supplementary material for: Understanding and predicting COVID-19 clinical trial completion vs. cessation
Source: PLoS One. 2021 Jul 12;16(7):e0253789. doi: 10.1371/journal.pone.0253789 (PMC8274906; doi:10.1371/journal.pone.0253789)
Supplement: Attachment — Submitted filename: COVID_19_Clinical_Trials_PLOS_One_Revision_Sumary___R1_.pdf [file pone.0253789.s002.pdf]

Re: Understanding and Predicting COVID-19 Clinical Trial Completion vs.Cessation

Revision Summary

We thank the Editor and the reviewers for their critiques and insights, which have been carefully taken into consideration in the revision. In order to address the reviewers' concerns, we have conducted a thorough revision of our manuscript.

The major changes of this revision are summarized as follows:

(1) We added additional information to the Introduction section and ensured a clear statement of our study objective.
(2) A new subsection **Related Work** was added to the Introduction to discuss related research and compare the existing research to our current study.
(3) Table 1 was added in subsection **Related Work** to present a graphical summary and comparison between existing methods and proposed research.
(4) We have added a subsection **Contribution** to the Introduction to summarize our current study's contribution. The main contributions of our study are as follows:

—COVID-19 clinical trial benchmark: Our research delivers a COVID-19 benchmark for clinical trial completion *vs.* cessation study. The benchmark, including features and supporting documents, are published online for public access.
—Clinical trial features: Our research proposes the most extensive set of features for clinical trial reports, including features to model trial administration, study information and design, eligibility, keywords, and drugs *etc.*. In addition, our research also uses embedding features to model unstructured free texts in clinical trial reports for prediction.
—Predictive modeling of COVID-19 trials: Our research is the first effort to model COVID-19 clinical trial completion *vs.* cessation. By using ensemble learning and sampling, our model achieves over 0.87 AUC scores and over 0.81 balanced accuracy for prediction, indicating high efficacy of using computational methods for COVID-19 clinical trial prediction.

(5) We introduced Friedman statistical tests to determine the statistical difference between ensemble classifiers tested over the different combinations of features. To give background information on the statistical test, a subsection **Statistical Tests** was added to the **COVID-19 Clinical Trial Prediction** section to explain the Friedman test and Nemenyi post hoc test
(6) Results from Friedman test and Nemenyi post hoc test are reported in **COVID-19 Clinical Trial Prediction** section. Figure 15 was added to show the critical difference diagrams for ensemble models' Balanced accuracy, F1, and AUC scores.
(7) To simplify the results, we only display average performance comparisons between single models *vs.* ensemble models using all features.
(8) The last paragraph of the result section presents an itemized list to clearly summarize the major findings from our experimental results.
(9) We have gone carefully through the paper and fixed spelling and grammatical errors.

In the following, we provide detailed responses to all the comments from the reviewers. The text in italics is taken verbatim from the reviewers' comments, and the roman text is our response.

Yours Sincerely,
Magdalyn Elkin and Xingquan Zhu

Response to Reviewer 1

(1) *In Introduction section, the drawbacks of each conventional technique should be described clearly.*

Response: Following the reviewer's suggestions, we have added a subsection **Related Work** to the Introduction to describe the drawbacks of conventional methods such as manual examination and categorization of terminated clinical trials. These are as follows:

—While manual examination can determine primary reasons for trial termination and the percentages of trials that terminate for those reasons, there is little insight on what specific factors lead to termination.
—Often the reason why a trial was terminated is not listed [Pak et al. 2015]
—Previous studies focused on all clinical trials in the ClinicalTrials.gov database. This provides little specific knowledge on the challenges that COVID-19 clinical trials face.

(2) *Introduction needs to explain the main contributions of the work more clearly.*

Response: Following the reviewer's suggestions, a new subsection **Contribution** was added to the Introduction to address the main contributions of our research. In summary, the main contribution of our research is threefold:

—COVID-19 clinical trial benchmark data set.
—Extensive set of features including trial administration, study information and design, eligibility, keywords, drug interventions as well as embedding features to model unstructured free texts.
—Predictive modeling of COVID-19 completion *vs* cessation.

(3) *The authors should emphasize the difference between other methods to clarify the position of this work further.*

Response: The difference between the current work and other research studies is detailed in the section **Related Work** as well as depicted in Table 1.

(4) *The Wide ranges of applications need to be addressed in Introductions*

Response: The ranges of applications is addressed in the introduction in the last two paragraphs, mainly these are as follows:

—Predictive studies are helpful for stakeholders to plain their resources, reduce cost and minimize time of clinical study
—Computational approaches allow for a deeper understanding and prediction of factors associated to successful COVID-19 clinical trials

(5) *The objective of the research should be clearly defined in the last paragraph of the introduction section.*

Response: The last paragraph of the Introduction, prior to the **Related Work** section, defines the objective of our study. The objective is as follows:

—Create features to model COVID-19 clinical trial reports.
—Using feature selection to determine features are highly associated to trial completion *vs* cessation.
—Use machine learning algorithms and ensemble learning to predict completion *vs.* cessation of COVID-19 clinical trials.

(6) *The authors can cite the following references.*
Analysis of dimensionality reduction techniques on big data.
Deep learning and medical image processing for coronavirus (COVID-19) pandemic: A survey.
An Incentive Based Approach for COVID-19 planning using Blockchain Technology.

Response: We have included a citation [Bhattacharya et al. 2021] in the Introduction section.

Response to Reviewer 2

(1) *Please re structure the paper format*

Response: Following the reviewer's suggestions, we have restructured the paper format accordingly. Meanwhile, some tables do span a wide area of the paper, due to the nature of the table content. We believe that the typewriting office will finally correct such format, if the paper is accepted for publication.

(2) *What are the limitations of the existing literature?*

Response: In the revision, we have added a subsection **Related Work** to the Introduction to discuss the limitations of the existing research. These are summarized as follows:

—While manual examination can determine primary reasons for trial termination and the percentages of trials that terminate for those reasons, there is little insight on what specific factors lead to termination.
—Often the reason why a trial was terminated is not listed [Pak et al. 2015]
—Previous studies focused on all clinical trials in the ClinicalTrials.gov database. This provides little specific knowledge on the challenges that COVID-19 clinical trials face.

(3) *List out the main contributions of the current work.*

Response: A new subsection **Contribution** was added to the Introduction to address the main contributions of the work. The main contributions are as follows:

—COVID-19 clinical trial benchmark data set.
—Extensive set of features including trial administration, study information and design, eligibility, keywords, drug interventions as well as embedding features to model unstructured free texts.
—Predictive modeling of COVID-19 completion *vs.* cessation.

(2.5) *Visit this profile and cite couple of related papers: https://scholar.google.com/citations?user=dz4QKZIAAAAJhl=en.*

Response: Following the reviewer's suggestions, we have cited one most relevant article [Thilakarathne et al. 2020] in the revision.

(4) *There are many typos and grammatical errors in the paper. The authors have to carefully proofread the paper to address them.*

Response: We have gone through the paper and corrected the typos and grammatical errors.

(5) *Summarize the related works section with a table.*

Response: Following the reviewer's suggestions, we have added Table 1 in **Related Work** to summarize related work, and compare related research with our current method.

(6) *Present a detailed analysis of the results obtained.*

Response: Following the reviewer's suggestions, We simplified the results presented and introduce statistical tests to have a more concise analysis of our results. These changes include the following:

—We report average performance comparisons between single models *vs* ensemble models using all features.

—We perform Friedman test and Nemenyi post hoc test to show the significant difference between ensemble classifiers on the different combinations of features.

—From statistical tests, we determine that there is a statistical difference between the four classifiers in Balanced Accuracy, F1 and AUC. The Nemenyi post hoc results are reported in critical difference diagrams in Figure 15. These results confirm that Random Forest has the best ranking out of all features and Neural Network as the worst ranking out of all features.

In the revision, we have included a breakdown of our major findings in the last paragraph of the results section. These are as follows:

—Feature selection does not improve AUC scores for trial prediction.

—Ensemble models show an improvement in F1 and balanced accuracy scores; a minimal improvement in AUC scores and a decrease in accuracy scores.

—Random Forest has the best performance and neural network has the worst performance.

—Random Forest ensemble models achieve 81% in balanced accuracy and 87% in AUC, indicating our model can achieve satisfactory accuracy for completion *vs.* cessation COVID-19 clinical trial prediction.

(7) *Discuss about the limitations and future scope of the present work.*

Response: In the revision, we have included a brief discussion of the limitations and future scope of our research in the Conclusion section.

A major limitation is the small number of clinical trial reports which meet the inclusion criteria for our study (Clinical trial status of Completed, Terminated, Suspended or Withdrawn). As more COVID-19 clinical trials are completed or stopped prematurely, the dataset will naturally grow. However, future work could incorporate ongoing COVID-19 clinical trials to create a semi-supervised model which may improve predictive model performance.

REFERENCES

BHATTACHARYA, S., REDDY MADDIKUNTA, P. K., PHAM, Q.-V., GADEKALLU, T. R., KRISHNAN S, S. R., CHOWDHARY, C. L., ALAZAB, M., AND JALIL PIRAN, M. 2021. Deep learning and medical image processing for coronavirus (covid-19) pandemic: A survey. *Sustainable Cities and Society 65*, 102589.

PAK, T. R., RODRIGUEZ, M. D., AND ROTH, F. P. 2015. Why clinical trials are terminated. Available from: `https://www.biorxiv.org/content/10.1101/021543v1`.

THILAKARATHNE, N., KAGITA, M. K., AND GADEKALLU, T. 2020. The role of the internet of things in health care: A systematic and comprehensive study. *International Journal of Engineering and Management Research*.
